# Microbiota and Myopericarditis: The New Frontier in the Car-Diological Field to Prevent or Treat Inflammatory Cardiomyo-Pathies in COVID-19 Outbreak

**DOI:** 10.3390/biomedicines9091234

**Published:** 2021-09-16

**Authors:** Andrea Piccioni, Angela Saviano, Sara Cicchinelli, Laura Franza, Federico Rosa, Christian Zanza, Michele Cosimo Santoro, Marcello Candelli, Marcello Covino, Giulia Nannini, Amedeo Amedei, Francesco Franceschi

**Affiliations:** 1 Department of Emergency Medicine, Fondazione Policlinico Universitario, Università Cattolica del Sacro Cuore, 00168 Roma, Italy; andrea.piccioni@policlinicogemelli.it (A.P.); sara.cicchinelli@policlinicogemelli.it (S.C.); cliodnaghfranza@yahoo.it (L.F.); federosa1991@gmail.com (F.R.); michelecosimo.santoro@policlinicogemelli.it (M.C.S.); marcello.candelli@policlinicogemelli.it (M.C.); marcello.covino@policlinicogemelli.it (M.C.); francesco.franceschi@policlinicogemelli.it (F.F.); 2 Department of Anesthesia, Critical Care and Emergency Medicine, Pietro and Michele Ferrero Hospital, Foundation Nuovo-Ospedale Alba-Bra, 12060 Verduno, Italy; christian.zanza@live.it; 3 Department of Experimental and Clinical Medicine, University of Firenze, 50134 Firenze, Italy; giulia.nannini@unifi.it (G.N.); amedeo.amedei@unifi.it (A.A.)

**Keywords:** microbiota, myocarditis, COVID-19, emergency department

## Abstract

Myopericarditis is an inflammatory heart condition involving the pericardium and myocardium. It can lead to heart failure, dilated cardiomyopathy, arrhythmia and sudden death. Its pathogenesis is mainly mediated by viral infections but also can be induced by bacterial infections, toxic substances and immune mediated disorders. All these conditions can produce severe inflammation and myocardial injury, often associated with a poor prognosis. The specific roles of these different pathogens (in particular viruses), the interaction with the host, the interplay with gut microbiota, and the immune system responses to them are still not completely clear and under investigation. Interestingly, some research has demonstrated the contribution of the gut microbiota, and its related metabolites (some of which can mimic the cardiac myosin), in cardiac inflammation and in the progression of this disease. They can stimulate a continuous and inadequate immune response, with a subsequent myocardial inflammatory damage. The aim of our review is to investigate the role of gut microbiota in myopericarditis, especially for the cardiovascular implications of COVID-19 viral infection, based on the idea that the modulation of gut microbiota can be a new frontier in the cardiological field to prevent or treat inflammatory cardiomyopathies.

## 1. Introduction: Microbiota and the “Heart–Gut Axis”

The growing interest in the study of the human microbiota has led to the evidence that many organs, that were once supposed to be sterile, also host their resident gut microbial communities. Interestingly, microorganisms residing in different body districts are not compartmentalized but crosstalk, through to the release of endotoxins and metabolites, that can then reach other microbial populations through the bloodstream [1]. The microbiota is the group of microbe populations living in the human body, and it includes bacteria, archaea, and viruses [1]. The gut microbiota is made up of the largest number of microbes, consisting of over one thousand resident microorganisms. Among bacteria, the main phyla are Firmicutes, Proteobacteria, and Bacteroidetes. While obtaining their habitat and nourishment from the host, these microbes protect the host from other pathogens, preventing infections. Indeed, their interaction with the intestinal surface, which constitutes the microbial niche, works as a physical barrier, increases competition for nutrients, helps produce antimicrobial peptides, and modulates immune cell function both in a pro- and anti-inflammatory fashion. All these effects have an impact on the immune system and consequently affect the susceptibility and the clinical course of many diseases.

Gut dysbiosis consists of an imbalance in the composition of the microbiota. It can lead to cardiovascular diseases [2,3]. At the same time, the presence of cardiovascular disorders can be responsible for gut dysbiosis. In fact, when cardiovascular function is impaired, the blood supply of the gut is not sufficient to maintain a health gut barrier, thus promoting a “leaky gut” situation [4].

More specifically, the “gut–heart axis” relies both on metabolism-dependent and metabolism-independent processes. In metabolism-dependent pathways, the gut microbiota acts like an endocrine organ that generates bioactive metabolites, including the trimethylamine/trimethylamine N-oxide (TMAO), short-chain fatty acids (SCFA), and primary and secondary bile acids (BA). In metabolism-independent mechanisms, impaired heart function contributes to splanchnic circulation congestion, bowel wall edema, and altered intestinal barrier, resulting in bacterial translocation, passage of bacterial products in the bloodstream, and subsequent pro-inflammatory state. Besides negatively influencing the function of many other organs, these changes may also worsen the heart function itself, in a vicious circle [2].

There is evidence of the role of the microbiota in atherosclerotic disease, coronary artery disease, and myocardial infarction pathogenesis. In particular, the atherosclerotic plaque contains DNA of the oral and the gut bacteria, and people with unstable plaques present reduced levels of anti-inflammatory peptides producing bacteria in the stool [5]. Moreover, high levels of TMAO have been associated with vulnerable coronary plaque, plaque rupture, and long-term risks of incident cardiovascular events in patients with acute coronary syndrome [6].

In the light of the described evidence, the modulation of microbial gut communities is an emergent topic to offer therapeutic strategies in heart disease. For example, some authors reported that—in animal models—the administration of antibiotics or probiotics reduces the extension of myocardial infarction size [7,8]. In an experimental study conducted on rats with coronary artery disease, the administration of the probiotic *Lactobacillus rhamnosus GR-1* for 6 weeks significantly improved the systolic and diastolic function of left ventricle, with benefits postinfarction heart remodeling and heart failure [8]. This probiotic was chosen owing to its ability to modulate the immune system via the gut, thus confirming the strict communication between gut and heart, through immune-mediated mechanisms [8]. In addition, this *Lactobacillus* was proved to contribute positively to the cardiac metabolism profile, preserving the cardiac cells taurine content [8]. Taurine is an amino acid abundantly expressed in the heart, with a role in heart failure and ventricular function. In the same way, *Lactobacillus rhamnosus GR-1* promotes gut health and reduces the concentration of some adipokines as leptin, providing protective effects on cardiac tissue [8].

Similar results were obtained with the use of a probiotic juice composed *Lactobacilli* (i.e., *Lactobacillus Plantarum*) and *Bifidobacterium* (i.e., *Bifidobacterium Lactis*) that also decreased the levels of leptin achieving the same benefits in terms of myocardial protection [7].

The administration of antibiotics in a study conducted on rats with coronary artery disease was demonstrated to have a protective effect on cardiac cells, too [7]. In fact, the treatment with vancomycin reduced the levels of the cytokine leptin with favorable effects on cardiac cells. Moreover, the antibiotic vancomycin given orally with water in these animals was tested to be effective in achieving cardio-protection. The same antibiotic introduced directly into the coronary circulation did not achieve the same result. Authors hypothesize that the intravenous administration did not modulate the gut microbiota, differently from the orally administration that reached the intestine [7,8]. This underlines that there is a link between gut microbiota and cardiovascular disease with the possibility of gut microbiota to have an effect on cardiac protection. Moreover, the protective cardiac effect obtained after two days of oral therapy ended three days after stopping the treatment and more studies are needed to explore this issue [7].

Similarly, in animal models of experimental autoimmune myocarditis, an increase in microbial diversity and richness has been observed [9]. Interestingly, it was also observed that in different mice with different gut microbiota colonization profiles, susceptibility to autoimmune myocarditis varied immensely [10].

The reduction of dysbiosis through antibiotics or other modern technique as fecal microbiota transplantation is able to modify the microbiome profiles in myocarditis mice, decreasing the inflammatory cardiac processes and the fibrotic cardiac dysfunction, the myocardial injury [10].

To date, much more scientific evidence is emerging on the strong connection between the profile of the gut microbiota and cardiac health and diseases, but other clinical research studies are needed to understand this field and its application in clinical practice more clearly.

In addition, the spread of COVID-19 disease worldwide has prompted investigation into how to deal with viral infections that also have the potential of changing the composition of the gut microbiota [11], influencing cardiac health [10].

Studies about COVID-19 reported modifications in gut microbiota composition induced by the viral infection [12,13,14,15] and in immune system functions [16,17,18,19,20]. In some cases, studies reported cardiac involvement with a cardiac disease (as inflammatory cardiomyopathies, myocarditis, and pericarditis) as a manifestation of COVID-19 infection [21,22,23,24,25,26,27,28,29,30,31,32,33,34,35,36,37,38,39], too. To date, research to better clarify these topics is ongoing.

## 2. Materials and Methods 

This narrative review included studies published in any language over the last 10 years, on the topic of microbiota and cardiovascular diseases, mainly focusing on cardiomyopathy and myopericarditis. In addition, we extended the search to include papers on COVID-19 and its impact on the development of myopericarditis, due to the presence of literature reports that underlined a connection between them. We searched systematic reviews, clinical trials, observational studies (longitudinal, cross-sectional, case–control, case series). We extracted data on the base on period of research, type of study, title, abstract. We searched on Web of Science^®^, PubMed^®^, UptoDate^®^, Cochrane^®^. No ethical approval was required to perform this review. The principal words we searched for were: microbiota AND cardiovascular diseases, microbiota AND COVID-19 AND/OR myocarditis, pericarditis AND microbiota, COVID-19 AND myopericarditis, Fecal transplantation AND/OR cardiovascular diseases, Dysbiosis AND viral infections AND cardiomyopathies.

## 3. Microbiota and COVID-19

In the process of viral invasion, the intestinal microbiota plays a key role, acting as a barrier, interacting directly or indirectly with the virus, and stimulating the innate and adaptive immune responses. Viral infections also have the potential of changing the composition of the gut microbiota [11]. Indeed, during COVID-19, modifications in microbiota composition have been reported [12,13,14,15]. Patients with COVID-19 had a higher number of opportunistic pathogens and depletion of beneficial commensals. On the one hand, the baseline abundance of some pro-inflammatory bacteria such as *coprobacilli* and *Clostridiodes* was higher and correlated with a more severe disease course. On the other hand, anti-inflammatory bacteria like *Faecalibacterium prausnitzii*, *Eubacterium rectale* and bifidobacteria were underrepresented. Interestingly, some bacteroides (*B. dorei*, *B. thetaiotaomicron*, *B. massiliensis*, and *B. ovatus*) downregulate the expression of angiotensin-converting enzyme 2 (ACE2), reducing the possibility of virus-entry thus causing a lower SARS-CoV-2 viral load in fecal samples of patients [13,14,15].

Liu et al. studied the effect of fecal microbiota transplantation in 11 patients who recovered from COVID-19 and who had suffered with gastrointestinal symptoms, gut dysbiosis and alteration of their immune status. Fecal microbiota transplantation restored gut microbiota and alleviated gastrointestinal disorders, but also had an impact on the immune system as demonstrated by changes in the peripheral lymphocyte subset [16]. In addition, there was also an increase in *Bifidobacterium* and *Faecalibacterium* and restored Actinobacteria and Proteobacteria. The modulation of gut microbiota was also proven to reduce COVID-19 disease severity, with beneficial effects on gastrointestinal symptoms, too [16]. Additionally, other study groups are investigating the effects of gut microbiota modulation during COVID-19 infection. In particular, studies are focusing on the effects of microbiota in modulating the immune system of the host, particularly in terms of progression of cytokine storm. It is well known that gut microbiota strictly interacts with host immune system. Their relationship is complex and dynamic. Factors such as viral infections can modify this balance, triggering inflammatory and immune diseases [14]. However, many studies focusing on the interaction between immunity and microbiota have tried to sequence and characterize the microbiome’s profile and to investigate on gut–microbiota modulators [13,14,15,16]. Another aspect that has been investigated is the role of KB109, a synthetic peptide, that can act as a microbiota modulator. In two trials it is currently being investigated whether or not it could significantly and positively impact microbiota composition, as a positive effect of COVID-19 disease [17,18,19].

## 4. Inflammatory Cardiomyopathies and COVID-19

The first reports in scientific literature regarding cardiac involvement during SARS-CoV-2 infection go back to the first months of the pandemic outbreak.

Many authors have described cases of patients with COVID-19 and inflammatory cardiomyopathies, mostly myocarditis [20,21,22,23,24,25,26,27,28,29,30,31,32,33,34,35,36,37] but also pericarditis [38,39,40,41,42,43]. In some cases, the cardiac disease was the first manifestation of the viral infection [21,29,39]. In other patients it was a life-threatening early complication, [24,25,31,38,44], or a fatal one [20]. Moreover, it could clinically display after infection recovery [30,32,35,37,45], or some-times remain subclinical, being detected only after cardiac imaging taking place for other reasons [46]. Manifestation could be cardiac tamponade or constrictive pericarditis, overall varying widely.

Acute myocardial dysfunction during the early course of COVID-19 has been retrospectively reported in 16–36% of patients [47,48]. While the etiology is unclear, it has been suggested that cardiac damage in COVID-19 could be attributed to stress-induced cardiomyopathy and immunological and microvascular damage [49].

The virus can indeed directly infect cardiac cells, entering in the endothelial blood vessels and activating immune response with endothelitis. Moreover, it can dysregulate hormonal pathways and activate proinflammatory responses with the release of many cytokines and the involvement of neutrophils, macrophages, platelets, and lymphocytes, thus provoking a prothrombotic state and a predisposition for clotting. This can happen both in the cardiac micro- and macro-vessels, with potentially many complications [49].

Moreover, it can dysregulate hormonal pathways and activate proinflammatory responses, with the release of many cytokines and the involvement of neutrophils, macrophages, platelets, and lymphocytes, thus provoking a prothrombotic state and a predisposition for clotting. This can happen in the cardiac micro and macrovessels. There are many different possible consequences, which range from the above discussed myocarditis and pericarditis, but also sudden arrythmias, heart failure and cor pulmonaris, plaque rupture. Another manifestation, closely associated with stress-induced mechanisms, is Takotsubo syndrome [49].

Post-mortem examination of hearts revealed potentially COVID-19-related cardio-vascular histopathologic findings, such as macro or microvascular thrombi, inflammation, and presence of intraluminal megakaryocytes. Even if these manifestations have been reported in almost half of autopsies (47.8%), functionally significant myocarditis was identified in only 2% of all cases [50]. Interestingly, when analyzing myocardial biopsies of patients with myocarditis/myopericarditis, no evidence of intracellular virus was reported [51]. Yet, some authors reported the presence of SARS-CoV-2 mRNA in the endomyocardial biopsies of patients with clinically suspected myocarditis, both testing positive [52] or negative for COVID-19 by nasopharyngeal swab [53]. SARS-CoV-2 particles were found in the cardiac macrophages or in the endothelial cells [54] but not directly in cardiomyocytes; some authors have thus suggested that the cardiovascular damage was caused by overall immune activation, rather than by direct viral induced damage [55].

The above discussed evidence has contributed to identifying and describing the “acute COVID-19 cardiovascular syndrome” (ACoVCS) in adults [56]. Yet, even though it has been identified as a clinical entity, its pathogenesis still remains largely unknown. Molecular mimicry and endothelial dysfunction have been hypothesized, but more studies are needed to confirm these results [51].

Even if our review focuses on adult patients, it is interesting to note that the ACoVCS presents some similarities with the “multisystem inflammatory syndrome in children” (MIS-C) described by Most, that has been reported in children with SARS-CoV-2 infection [56]. At a cardiac level, the MIS-C can present itself with coronary dilatation, which resembles Kawasaki disease [55,56]. Other manifestations can be elevated troponin, cardiogenic shock, and reduced biventricular function. In this study, all children tested negative for SARS-CoV-2 by polymerase chain reaction (PCR) test, but they also had specific IgG antibodies. The authors thus concluded that MIS-C could depend on a post-infectious inflammatory state that occurs several weeks after a primary infection [56]. 

There is much more evidence on the association between COVID-19 and cardiovascular complications, such as myocarditis, pericarditis, fulminant myocarditis with arrhythmias, as described above, but there is a gap of knowledge in understanding the different pathogenic mechanisms [9]. Fox et al. analyzed myocardial biopsies of COVID-19 patients with myocarditis and found an increased number of CD68 + cells (that indicate monocyte/macrophage lineage), compared to myocarditis which are not associated to COVID-19 infection [57]. Similar observations have been reported by other authors as well [52]. The significance of these data has not been clarified yet and the possible association with prognosis and mortality is unclear. Lethal complications of myocarditis (such as end stage heart failure, cardiogenic shock, etc.) have also been observed in patients with COVID-19 infection [51]. Sawalha et al. noticed that cardiac tamponade was present in 20% of echocardiograms in patients with COVID-19 infection. Moreover, patients who died usually also had a serious acute respiratory distress syndrome as well [58]. 

When considering clinical presentation, ACoVCS resembles other myocarditis. Indeed, patients mostly report oppressive chest pain or dyspnea, with an electrocardiogram that can be normal or may show alterations like ST-elevation or T-inversion. Troponin can be elevated above the normal value but does not typically present a significant delta between tests. Echocardiography can reveal a normal or reduced ejection fraction. Concerning the imaging of ACoVCS, as for other myocardiopathies, cardiac magnetic resonance seems to be the best diagnostic option [46,59]. Esposito et al. found myocardial edema and wall thickening in eight patients with clinical suspect of myocarditis who underwent a cardiac MRI [59]. Authors concluded that inflammation was a substrate of myocardial injury in patients with SARS-CoV-2 infection. Further studies are necessary to fill the gaps and to solve the controversies as regards the direct and indirect effects of COVID-19 disease on myocardial cells and to better understand the cardiovascular consequences of its infection (Table 1).

## 5. Inflammatory Cardiomyopathies and Microbiota

Myopericarditis is an inflammatory condition, in which a pericarditis and a myocarditis coexist [60]. The clinical presentation is often not specific; patients can experience chest pain and dyspnea and have an electrocardiogram with segment ST-elevation. For the diagnosis it is essential to rule out other etiologies, such as obstructive coronary disease. An echocardiogram can also be very useful in this context, as it can rule out some other causes of the symptoms experienced by the patient. The gold standard, in clinical practice, for the diagnosis is the cardiac magnetic resonance, which is useful for the identification of myocardial inflammation and damage [9]. A biopsy is required in selected cases, if the MRI is not able to provide a definitive diagnosis [61]. Myocarditis can also evolve into inflammatory cardiomyopathy, which is a medical emergency due to the high mortality risk. Genetic and environmental factors may predispose to progression and complications [62].

In most cases, myopericarditis is caused by cardiotropic viruses [63,64], but bacterial infections, toxic substances and immune-mediated diseases have also been linked to the development of the condition [9]. In the case of viral infection, authors differentiate between a “cardiotopic effect” and a “lymphotropic effect”. In the first case there is a direct, virus-induced, cardiac damage. In the second case, instead, there is an inflammatory cardiomyopathy, associated to the activation of the immune response, promoted by the virus, through a number of different immunologic pathways [9].

With regard to the lymphotropic mechanisms, auto-inflammatory disorders are also a common manifestation and, overall, even if it is not the only and direct cause, autoimmune response is an important driver of heart disease progression.

In this context, studies on animal models focused on understanding the “cross-mimicry” and the “cross reactivity” phenomena, and on the link between gut bacterial and cardiac inflammation [65].

For example, Gil-Cruz et al. have observed that commensal gut bacteria like Bacteroides (*vulgatus*, *diastonis*, *theta*, *thetaiotaomicron*, *faecis*) or Enterococcus cloacae can produce myosin-like peptides. These peptides can cross-activate T-lymphocytes (Th1 and Th17 more specifically), promoting cardiotoxic damage [62]. These events lead to myopericarditis and dilative inflammatory cardiomyopathy [65].

Besides “cross-mimicry”, another mechanism has also been implicated in the development of the disease, consisting of the creation of a pro-inflammatory milieu promoted by dysbiosis. Indeed, while it is unlikely for these pathogens to directly damage the heart—even though bacterial translocation in the bloodstream has been described—it is likely that modifications in its composition can create an inflammatory environment, which can then precipitate the development of inflammatory cardiomyopathies [66].

A study by Xiao-Fan et al. focuses on dysbiosis related to the onset of experimental autoimmune myocarditis (EAM) in murine models, in which it was shown that the progression of the disease is associated with an increase in the Firmicutes/Bacteroidetes ratio [4]. In another study, the correlation between myocarditis and the progression towards its complication, inflammatory cardiomyopathy, was analyzed [67]. It was observed that the progression also depends on the inflammatory stimulation by a species of Bacteroides in genetically pre-disposed individuals.

In the light of this evidence, the modulation of gut microbiota composition can be a potential treatment for acute cardiomyopathies. Mice with different microbiome patterns develop different forms of myocarditis, and antibiotics administration changes the progression of the disease [10]. Moreover, studies conducted on mice demonstrated that fecal transplant is effective in treating autoimmune myocarditis. Indeed, fecal microbiota transplantation led to reduction in myocardial injury and of inflammatory infiltration, with a decrease in IFN-γ gene expression in the heart tissue and CD4 + IFN-γ + cells in the spleen. Additionally, the ratio between Firmicutes and Bacteroidetes was restored [4].

With regard to pericarditis, not many studies have been conducted on the relationship between the microbiota and their treatment. However, there is some evidence regarding the connection between Bacteroides and the onset of pericarditis. In particular, Bacteroides fragilis species can become pathogenic and give rise to a series of diseases including pericarditis [68]. For this reason, we believe that although there is currently not much evidence in this regard, in the future studying the relationship between microbiota and pericarditis will provide new therapeutic strategies to be used to improve patients’ outcomes.

## 6. Final Remarks

Cardiovascular diseases account for 31% of all global deaths and are the largest contributor to mortality and morbidity worldwide. With more and more developments in scientific research, the crucial and impacting role of gut microbiota in these pathologies has been proven and well demonstrated. Some data suggest that the gut microbiota could be able to improve prognosis of cardiovascular diseases, reducing several risk factors and its modulation is expected to become a new therapeutic approach [69].

Viral infections are known to be able to alter the equilibrium in the gut microbiota. COVID-19 infection is also among the infections able to determine dysbiosis, which might also partly explain the correlation between myopericarditis and COVID-19 infection. Indeed, this disease caused an enrichment of opportunistic pathogens and depletion of beneficial bacteria. It is already documented that patients with cardiac failure show reduced capacity to absorb substrates and an increase in intestinal permeability, often characterized by reduced diversity of gut microbiota and imbalances between bacterial families [70,71,72]. Considering that at least in some viral infection processes, the intestinal microbiota seems to have a key role, it is more and more evident that viral infections may be able to change the microbiota composition. Moreover, observing that the gut microbiota seems to modulate the responses at least during some respiratory infections, this also could be the case for COVID-19 [73]. Ten gastrointestinal symptoms accompany influenza infection, and, in animal models, respiratory infection has been shown to alter the microbiota [73,74,75,76]. Considering that the gut microbiota modulates the responses during respiratory infections, this also could be the case for COVID-19. Recent data demonstrated that COVID-19 patients have significant differences in gut microbiota composition and on the interaction between microbiota and host cells [73]. On the other side, as described in the introduction section, gastrointestinal dysbiosis can lead to cardiovascular diseases [2,3,77] and cardiovascular impairment can contribute to maintain gut microbiota dysbiosis [78]. In particular, many studies have described cases of patients with COVID-19 and inflammatory cardiomyopathies. Our review took into consideration the hypothesis that this triad, cardiomyopathies–COVID-19 infection–microbiota, is strongly connected. Recent evidence, both from animal and human studies, supports the idea that gut microbiota dysbiosis can play a role in cardiovascular diseases [79]. In particular, the dysbiosis can be responsible of cardiotoxicity and phenomenon of cardiac inflammation. Therefore, determining the COVID-19 infection a functional and taxonomic reshaping of gut microbiota, it is very likely the cardiovascular repercussions; large epidemiological studies and animal models are needed to better clarify this important correlation. Finally, modulating the gut microbiota could provide a favorable impact on the course of these conditions, but it is reasonable to say that more studies (animal models, but even more so clinical trials) are required to test the efficacy of therapeutic intervention on gut microbiota in cardiovascular diseases and employ them in clinical practice.

## Figures and Tables

**Table 1:** Inflammatory cardiomyopathies and COVID-19.

Authors	Year	Results
Auer, J. et al. [20]	2020	A 42-years-old died of ventricular fibrillation on day 9 after ICU admission for COVID-19 pneumonia. Her exams showed increasing troponin and NT-proBNP. The autopsy revealed myocarditis.
Beşler, M.S. et al. [21]	2020	Myocarditis has been the first manifestation of SARS-CoV-2 infection in a 20-years old female with no prior cardiovascular disease.
Caballeros Lam, M. et al. [22]	2020	The paper reports two cases of people with SARS-CoV-2 infection and myocarditis. The first case is a young, asymptomatic, woman who tested positive in pre-partum screening. The delivery was regular, and she manifested chest pain a week later. Troponin was high and CMR revealed myocarditis. The second case is a 13-years-old boy without chest pain but with elevated troponin and NT-proBNP. The CMR detected myocarditis.
De Vita, S. et al. [23]	2020	A young woman presented to the ED with congestive heart failure-like symptoms one month after delivery. She reported flu-like syndrome few weeks before birth-giving. Her troponin, NT-proBNP, and d-dimer were high, and she tested positive for SARS-CoV-2 PCR. The chest CT-scan revealed pulmonary embolism, and CMR detected myocarditis with severe reduction of the ejection fraction (EF = 17%).
Doyen, D. et al. [24]	2020	A 67-years.old man was admitted to the ICU for a respiratory distress syndrome caused by COVID-19. His ECG showed diffuse T-inversion. Since his GRACE score was > 140, a coronary angiography was performed but resulted negative. CMR revealed myocarditis.
Irabien-Ortiz, A. et al. [25]	2020	A 59-years-old woman presented to the EC with flu-like symptoms and squeezing angina. The SARS-CoV-2 PCR was positive. A fulminant myocarditis was diagnosed because of high troponin, NT-proBNP, and D-dimer together with diffuse concave ST-segment elevation, pericardial effusion, and myocardial thickening with oedema and severe dysfunction with shock. During the hospitalization she was treated with immunoglobulins and steroids, required emergency pericardiocentesis and ECMO.
Jain, A. et al. [26]	2020	A 60-years-old male with SARS-CoV-2 infection was admitted to the ICU because of multi-organ failure (severe respiratory distress, cardiogenic shock and kidney failure). During the hospitalization a myocarditis was diagnosed. He required immunoglobulins and steroids, together with renal replacement therapy.
Kim, I.C. et al. [27]	2020	A 21-year- old female presented to the ED with febrile sensation, dyspnoea, and chest pain. The SARS-CoV-2 PCR was positive and the first exams (cardiac markers, electro- and echocardiography) were suggestive of myocarditis. The diagnostic suspicion was confirmed by a CMR.
Leutkens, J.A. et al. [28]	2020	A 79-years-old was admitted to ED because of dyspnoea and recurrent syncope. The first tests were negative. A contrast-enhanced chest CT-scan was performed to rule out pneumonia or pulmonary embolism but revealed ground-glass opacities. The naso-pharyngeal swab resulted positive. He was admitted to the ICU because of hemodynamic and respiratory worsening. Due to cardiac markers elevation a MRI was performed and revealed myocarditis.
Paul, J.F. et al. [29]	2020	A 35-yeras-old male, who tested SARS-CoV-2 positive, was admitted to the Cardiology ward because of fatigue and ECG changes in the precordial leads. The troponin was elevated. The CMR confirmed the myocarditis suspicion. He was treated with Ramipril and Bisoprolol, and recovered in few weeks.
Spano, G. et al. [30]	2020	Peri-myocarditis can be a delayed manifestation of COVID-19. A 49-year-old male who had a flu-like SARS-CoV-2 infection was admitted to the ED 6 weeks after his negative nasopharyngeal swab, because of heart-failure like manifestation. A cardiac MRE showed myocardial oedema and pericardial effusion consistent with peri-myocarditis. Other causes of peri-myocarditis have been ruled out.
Zeng, J.H. et al. [31]	2020	First reported case of myocardits in COVID-19 infection. A 63-year-old male admitted to the ICU for SARS-CoV-2 pneumonia showed high troponin levels and heart disfuction. Myocarditis was diagnosed, but despite progressive improvement of the ejection function the patient died because of the infection complications.
Bajaj, R. et al. [32]	2021	Myocarditis can be a delayed manifestation of COVID-19, due to a multisystem inflammatory syndrome occurring several weeks after SARS-CoV-2 infection. This condition can be difficult to identify for many reasons: negative RT-PCR testing at the time of the cardiac presentation, attribution of systolic impairment to pre-existing cardiac disease, high frequency of COVID-19-related acute myocardial injury (up to 40% of hospitalised patients have increased troponin concentrations), and difficulties obtaining complex or invasive diagnostic investigations in ICU patients during the pandemic.
Dahl, E.H. et al. [33]	2021	A 37-year-old, previously healthy man presented to the ED with fever, headache, and unilateral neck swelling. He tested positive for SARS-CoV-2, and his conditions deteriorated because of fulminant myocarditis. He recovered and, few week after, he represented for Bell’s palsy.
Laganà, L. et al. [34]	2021	This multicentre case series showed that patients with suspected myocarditis were older, had a higher frequency of previous cardiac disease, significantly more prolonged hospitalization, and a lower value of interleukin-6 than other COVID-19 patients.
Osorio Martinez, A. et al. [35]	2021	COVID-19 has been demonstrated to be a multisystemic inflammatory disorder. Patients with prior infection, and actual negative PCR for SARS-CoV-2, can display a variety of conditions including myocarditis, thyroiditis, and hepatitis, as described in this case report. For this reason, healed COVID-19 patients should receive a follow-up.
Sheikh, A.B. et al. [36]	2021	Beside early manifestations of COVID-19, long-term complications of the infections have been reported, involving many systems including both endocrine and cardiovascular systems. This interesting case report is about a young male with prior COVID-19, admitted to the hospital with myocarditis and diabetes insipidus.
Volis, I. et al. [37]	2021	A healthy young male, presenting for persistent fever 20 days after initial diagnosis of COVID-19 and after a clinical, and apparent laboratory, resolution of the original episode, showed ECG modifications. His troponin levels were raised and myocarditis was diagnosed.
Hua, A. et al. [38]	2020	A 47-year-old presented with chest pain and breathlessness. She was positive for COVID-19. ECG showed diffuse ST alterations, serum troponin was elevated, and a TTE showed cardiac tamponade. Due to refractory shock a pericardiocentesis was performed, with rapid improvement.
Kumar, R. et al. [39]	2020	A 66-year-old farmer presented pericarditis as the only evident manifestation of COVID-19. He complained of pleuritic chest pain. His ECG showed diffuse ST-elevation. CRP was elevated and troponin was normal. The TTE revealed an echo bright pericardium with no pleural effusion. He started oral colchicine two times per day for 2 weeks and was discharged on day 4.
Ortiz-Martinez, Y. et al. [40]	2020	A young internal medicine resident with COVID-19 presented to the ED for pleuritic chest pain. He was diagnosed with pericarditis with mild pericardial effusion and successfully treated with colchicine and ibuprofen.
Tung-Chen, Y. et al. [41]	2020	A young woman with was evaluated in the ED for paucisymptomatic COVID-19 with normal exams and thoracic echography. She was discharged but represented 6 days after with pleuritic centrothoracic chest pain that improved when sitting forward and worsened with the supine position. The ECG, then laboratory findings and the echocardiography, were consistent with pericarditis. She was successfully treated as an outpatient with colchicine.
Sandino Pérez, J. et al. [42]	2020	Transplanted patients are at higher risk of developing COVID-19 complications because of their immunodepression. The authors report the case of a kidney-transplanted 73-year-old male on tacrolimus and mycophenolate modfetil who was infected by SARS-CoV-2. After few days of hospitalization he complained of atypical chest pain with ECG evidence of precordial concave ST-elevation, raise of CRP with negative troponin. The TTE revealed pericarditis without pericardial effusion. He was successfully treated with colchicine.
Beckerman, J.K. et al. [43]	2021	A 55-year-old man experienced long hospitalization in the ICU for COVID-19, with many complications including acute kidney injury hemofiltration, a catheter-associated internal jugular vein clot, ventilator-associated pneumonia, *Enterococcus faecalis* bacteremia, *Clostridium difficile* colitis and multiple decubitus ulcers. On day 128 of admission, he developed constrictive pericarditis (documented with a CMR), requiring pericardiocentesis.
Salamanca, J. et al. [44]	2020	A previously healthy 44-year-old man, previously discharged from the ED with paucisymptomatic COVID-19, returned few days later with shock signs. The ECG showed a third-degree atrioventricular block, the TTE revealed a non-dilated but globally and severely dysfunctional left ventricle and the troponin was high. With suspected fulminant myocarditis (further confirmed with CMR), the patient required mechanical ventilation, a temporary pacemaker, and vasoactive/inotropic support. Despite these measures his conditions worsened and he required ECMO and an intra-aortic balloon pump. He improved in the following days.
Sardari, A. et al. [45]	2021	A 31-year-old internal medicine registrar presented with dyspnoea on exertion and low-grade fever. He had a history of COVID-19 pneumonia and was discharged 3 weeks previously. The ECG, CRP, and troponin were normal. The TTE revealed mild left ventricular dysfunction. Suspicions of active myocarditis were confirmed with CMR.
Ng, M.Y. et al. [46]	2020	Case series of 16 patients who recovered from COVID-19 who underwent CMR to assess for evidence of myocardial involvement or ongoing myocarditis. CMR was performed at a median of 56 days post-recovery.
Lala, A. et al. [47]	2020	The intention of the authors was to describe the degree of myocardial injury and associated outcomes in a large hospitalized cohort with laboratory-confirmed COVID-19 (*n* = 2736). Cardiovascular disease including coronary artery disease, atrial fibrillation, and heart failure, was more prevalent in patients with higher troponin concentrations, as were hypertension and diabetes.
Wei, J.F. et al. [48]	2020	The authors prospectively assessed the medical records, laboratory results, chest CT images and use of medication in a cohort of patients (*n* = 101) presenting to two designated COVID-19 treatment centres in Sichuan, China. Acute myocardial injury was present in 15.8% of patients, nearly half of whom had a hs-TnT value five-fold greater than the normal upper limit
Topol, E.J. et al. [49]	2020	Cardiac injury, as reflected by elevated concentrations of troponin, is common with COVID-19, and occurs in at least one in five hospitalized patients and more than half of those with preexisting heart conditions. Such myocardial injury is a risk factor for in-hospital mortality, and troponin concentration correlates with risk of mortality.
Halushka, M.K. et al. [50]	2020	The authors performed a literature review on 277 autopsied hearts of COVID-19 positive patients. Even if potentially COVID-19-related cardiovascular histopathologic findings, such as macro or microvascular thrombi, inflammation, or intraluminal megakaryocytes, were reported in 47.8% of cases, myocarditis was present in 20 hearts (7.2%), and most cases were likely not functionally significant ( <2%). In conclusion, COVID-19-related cardiac histopathological findings are common, while myocarditis is rare.
Mele, D. et al. [51]	2021	The review aimed to summarize the limited knowledge about mechanisms, prevalence, prognosis, diagnosis and therapy of myocarditis in the context of COVID-19.
Bearse, M. et al. [52]	2021	The study focuses on 41 fatal cases of COVID-19. In such patients, infection of the heart by SARS-CoV-2 is common but is often limited with only rare infected cells. When present, myocarditis is often a relatively late event in the terminal disease course. Cardiac infection by SARS-CoV-2 is associated with electrocardiographic changes. Non-biologic immunosuppression is associated with lower incidences of both myocarditis and cardiac infection.
Wenzel, P. et al. [53]	2020	First report of two patients with a history of COVID-19 in whom clinically suspected myocarditis was supported by endomyocardial biopsy with evidence of persisting cardiac SARS-CoV-2 mRNA
Farshidfar, F. et al. [54]	2021	COVID-19 can result in systemic inflammation affecting many systems, including the cardiovascular one. Cardiovascular complications include myocardial injury, myocarditis, acute myocardial infarction, heart failure, dysrhythmias, and venous thromboembolic events. Emergency clinicians should be aware of these cardiovascular complications when evaluating and managing the patient with COVID-19.
Mehta, P. et al. [55]	2020	Secondary haemophagocytic lymphohistiocytosis (sHLH) is an under-recognised, hyperinflammatory syndrome characterised by a fulminant and fatal hypercytokinaemia with multiorgan failure. A cytokine profile resembling sHLH is associated with COVID-19.
Most, Z.M. et al. [56]	2021	SARS-CoV-2 most often manifests with a pulmonary syndrome that evolves from viral pneumonia to an inflammatory mediated acute respiratory distress syndrome. Two less common clinical presentations of COVID-19 include multisystem inflammatory syndrome in children (MIS-C) and acute COVID-19 cardiovascular syndrome (ACovCS) in adults.
Fox, S.E. et al. [57]	2020	In a series of autopsies of fatal cases of COVID-19, cardiac findings included individual cell necrosis without lymphocytic myocarditis.
Sawalha, K. et al. [58]	2021	This systematic review focuses on fourteen cases of myocarditis/myopericarditis secondary to COVID-19. Guidelines for diagnosis and management of COVID-19 myocarditis have not been established: the use of glucocorticoids and other agents including IL-6 inhibitors, IVIG and colchicine is object of debate. The authors conclude that patients treated with steroids have better outcomes. However, until larger scale studies are avalaible, treatment should be individualized case-by-case.
Esposito, A. et al. [59]	2020	This paper reports the first series of patients with ACovS (*n* = 10; 8 females and 2 males; 52 ± 6 years of age) consecutively referred for CMR for suspected myocarditis between 15 March and 20 April, 2020, in 4 Italian university hospitals.

ACovS = acute COVID-19 cardiovascular syndrome; CRP = C-reactive protein; ECG = electrocardiogram; ECMO = extracorporeal membrane oxygenation; CMR = cardiac magnetic resonance; sHLH = secondary haemophagocytic lymphohistiocytosis; TTE = transthoracic echocardiogram.
